# Automated Detection of Synapses in Serial Section Transmission Electron Microscopy Image Stacks

**DOI:** 10.1371/journal.pone.0087351

**Published:** 2014-02-06

**Authors:** Anna Kreshuk, Ullrich Koethe, Elizabeth Pax, Davi D. Bock, Fred A. Hamprecht

**Affiliations:** 1 HCI/IWR, University of Heidelberg, Heidelberg, Germany; 2 Wheaton College, Wheaton, Illinois, United States of America; 3 Janelia Farm Research Campus, Howard Hughes Medical Institute, Ashburn, Virginia, United States of America; Virginia Tech Carilion Research Institute, United States of America

## Abstract

We describe a method for fully automated detection of chemical synapses in serial electron microscopy images with highly anisotropic axial and lateral resolution, such as images taken on transmission electron microscopes. Our pipeline starts from classification of the pixels based on 3D pixel features, which is followed by segmentation with an Ising model MRF and another classification step, based on object-level features. Classifiers are learned on sparse user labels; a fully annotated data subvolume is not required for training. The algorithm was validated on a set of 238 synapses in 20 serial 7197×7351 pixel images (4.5×4.5×45 nm resolution) of mouse visual cortex, manually labeled by three independent human annotators and additionally re-verified by an expert neuroscientist. The error rate of the algorithm (12% false negative, 7% false positive detections) is better than state-of-the-art, even though, unlike the state-of-the-art method, our algorithm does not require a prior segmentation of the image volume into cells. The software is based on the ilastik learning and segmentation toolkit and the vigra image processing library and is freely available on our website, along with the test data and gold standard annotations (http://www.ilastik.org/synapse-detection/sstem).

## Introduction

Synapses are the main conduit of information flow between neurons in the brain. If one considers brain wiring as a graph, neurons can be considered the vertices, and the synapses between neurons as edges. The emerging field of connectomics [1], [2] seeks to extract graphs from neural tissue and to determine the relationship between graph structure and the computational properties of neural circuits [3]–[7]. However, the anatomical substrate [8] of the connectivity graph presents significant technical challenges to image and trace [9], [10]. Each mammalian cortical neuron has a dendritic arbor, which usually receives the synaptic input to the cell, and an axonal arbor, which usually provides the output from the cell. Axons can be as fine as 100 nanometers (nm) in diameter, extend over hundreds of micrometers (*μ*m) even in local brain circuits. Along with the dendrites they almost completely pack the available brain volume in a tangled mass of “wires” called the neuropil. Chemical synapses form between axons and dendrites, and are characterized by thickened presynaptic (axonal) and postsynaptic (dendritic) membranes with a 10–20 nm cleft between them. Synaptic vesicles, 40–50 nm spheres packed with neurotransmitter, cluster about the presynaptic membrane. Within this simplified scheme, many variations exist throughout the nervous system which are important to neural circuits, such as axo-axonic synapses and electrical (gap junctional) synapses; here we focus on the subset of connections between neurons as defined by the chemical synapses between them.

Electron microscopy (EM) is capable of resolving all the structures needed to extract connectivity graphs from brain tissue. In recent years, a number of methods have been introduced for volume electron microscopy of brain tissue [11]. In general, these methods trade off ease of sample handling and data generation for resolution and imaging speed. Focused ion beam scanning electron microscopy (FIB-SEM) [12] offers high resolution, isotropic voxels (5×5×5 nm), but is limited to cubic volumes of about 50 *μ*m edge length, much smaller than is needed to contain complete neural circuits. Serial block face scanning electron microscopy (SBF-SEM) [13] and automated tape-collecting ultramicrotome scanning electron microscopy (ATUM-SEM) [14] provide lower resolution, anisotropic voxels (10×10×20 nm, and 4×4×30 nm, respectively), but have higher throughput than FIB-SEM. Serial section transmission electron microscopy (ssTEM)-based methods provide slightly lower resolution anisotropic data (4×4×40 nm) and have sample-handling disadvantages, but when imaging is done with a transmission electron microscope camera array (TEMCA) [5], offers substantially higher throughput. High throughput imaging is a great advantage when attempting to encompass millimeter-scale cortical volumes at nanometer resolution, since in this regime, the required time to image the target multi-terabyte (TB) volume otherwise becomes prohibitive. TEMCA would require 317 days to image a full mouse cortical column, compared to 505 days for ATUM-SEM or 818 days for SBEM [11].

Currently, extensive manual annotation is needed to extract even a small fraction of the wiring from available ssTEM image data. Automated synapse detection of chemical synapses (i.e. synapses whose effects are mediated by the release of neurotransmitter from vesicles at specialized release sites) offers an important challenge and tractable first step in automating extraction of neuronal connectivity data from electron microscopy volumes. This is the problem we aim to solve with this contribution.

Analysis of EM neural images provides a unique set of challenges, related to i) the similarity of local texture between different object classes, ii) high variability in shape and texture between objects of the same class and iii) enormous data volume. For data with anisotropic resolution, the problem is further aggravated by the changes in the appearance of thin or flat structures depending on their orientation relative to the cutting plane. Besides, the data acquisition process for serial section Transmission Electron Microscopy (ssTEM) involves tissue slices. Their manual or mechanical handling introduces image artefacts, such as rips and folds, and variations in image illumination and contrast. The independent slice images also require careful re-alignment for any automated processing to be possible.

### Previous Work

The problem of segmentation of EM neural images has received a lot of attention in recent years, which mainly concentrated on the task of the segmentation of cell membranes. Depending on the resolution of the data, such segmentation is either performed directly in 3D ([15]–) or, for ssTEM data, in 2D with subsequent z-axis linking ([20]–[23]).

For the task of fully automated synapse detection, several methods have recently been proposed for image volumes with *isotropic* resolution, acquired on Focused Ion Beam/Scanning Electron Microscopes. In [24], the first such algorithm was proposed, based on interactive pixel classification and 3D pixel features. This approach was further developed by Becker et al. in [25] by incorporation of local context features, specific for synaptic contact locations. Morales et al. in [26] propose a semi-automated method, which relies on pattern matching and, compared to [24] and [25], requires very substantial user input.

For ssTEM images of rabbit retina, Jagadeesh et al. in [27] propose a method, based on patch-based fused detection of vesicles, synaptic clefts and ribbons. The fusion can be performed either by simple concatenation of feature vectors or by using multiple kernel learning. A fast interest point detector is run first to ensure scalability to large datasets. The accuracy of the method on the detection task is 82%. While this method constitutes a very important contribution to the task of retinal connectome reconstruction, the 2D image features used by [27] are specific for ribbon synapses, which are characterized by electron-dense “ribbon” regions. These synapses are not present in the cortex.

Navlakha et al. in [28] approach the synapse detection problem from another side and develop a special tissue staining technique, which highlights synapses only. They also propose an automated detection method for data obtained in this way, which is unfortunately not at all applicable to conventional staining used for connectomics studies.

For the predominant use case of conventionally stained brain data, the first and, to the best of our knowledge, only method for automated synapse detection has been proposed by Mishchenko et al. in [29]. In this study, synapse detection served as a part of a large-scale semi-automated volume reconstruction effort and the synapse detection algorithm relied on a full prior segmentation of the volume into cells. Unfortunately, reliable automation of the cell segmentation task is not yet possible, despite impressive algorithmic advances of recent years [10].

Overall, we note that fully automated synapse detection is not yet available for EM data with *anisotropic* resolution. Neuroscientists are thus forced to resort to manual image annotation – a difficult and extremely time-consuming task. Our contribution intends to close exactly this gap and proposes an algorithm and a corresponding software tool to automatically detect chemical synapses in ssTEM image stacks of conventionally stained brain tissue. As for many computer vision applications, our aim is to get as close as possible to the performance of human experts. The precision and recall of the algorithm are thus evaluated against a “ground truth” dataset, created by consensus of human annotators. While these annotations have not been verified by additional imaging experiments to only contain true locations of synaptic contacts, we believe it to be a good representation of an expert manual annotation of the images.

### Proposed Approach

The algorithm is based on two stage learning: first, pixel classification and graph cut segmentation are used to find synapse candidates, which are then filtered by the second – object-level – classification step. Pixel and object-level classifiers are learned from training data supplied by human experts. Very sparse annotation is sufficient for the first stage; the second stage requires a few hundred binary labels.

Unlike the existing approaches for ssTEM data, we perform pixel classification directly in 3D and find that it greatly improves the precision of the method, by incorporating crucial axial context information.

After spatial regularization by an Ising model MRF, optimized by graph cut, we apply the second classification step with features based on summary statistics of the raw pixel values in the synapse candidate objects and in their neighborhoods. Inclusion of the neighborhood statistics allows to implicitly take the object context into account without designing specific features for this task.

We use the ilastik toolkit for pixelwise classification (http://www.ilastik.org), openGM optimization library for segmentation (http://hci.iwr.uni-heidelberg.de/opengm2/), the vigra image processing library for object classification(http://hci.iwr.uni-heidelberg.de/vigra/) and scikit-image toolkit for some of the object-level features (http://scikit-image.org/). All these libraries are free and open source and our scripts are also freely available on our website. Besides, we are planning to incorporate the synapse detection tool into the next release of ilastik and thus offer a unified GUI for interactive labeling on pixel and object level.

The modern EM setups allow for the acquisition of multiple Terabytes of data ([4]–[6]). While the available ground truth annotation limits our quantitative validation to 1GByte worth of data, we demonstrate in the Results section that our algorithm can scale to full connectomics datasets.

## Results

Quantitative validation of the algorithm has been performed on a test dataset with 238 manually annotated synapses (see Methods section for the details on data acquisition and ground truth generation). All time estimates were obtained on a desktop machine with an Intel Xeon ES-1650 CPU (12 cores, 3.20 GHz).

Pixel labels were provided interactively in ilastik, using a small stack of the data from [5], not overlapping with the test data. Three classes were used: one for synapses, one for membranes and one for the rest of the data. New labels were introduced until the Random Forest out-of-bag error on the training data stopped improving and the prediction of the algorithm on unlabeled synapses seemed visually stable [30]. In total, 4 slices were labeled very sparsely, in a manner similar to [24]. Pixel classification of a 1.2 GByte block took 132 minutes.

The result of pixel classification is a “probability map” of the volume, which for each pixel contains the probability of belonging to the defined classes. We detected the areas of high synapse probability by thresholding the probability maps of the synapse class at 0.5, running connected components analysis and discarding components of less than 100 pixels or more than 1000000 pixels in size. We perform the detection step in order to limit the more expensive segmentation step to areas mostly likely to contain synapses (see Methods section for more details). On a 1.2 GByte block, detection and extraction of the bounding boxes took 11 minutes with a single-core implementation.

To transfer from detection to synapse candidate objects, we applied the standard graph cut segmentation algorithm [31] on the probability maps. The unary potential for each pixel was assigned to its synapse probability, multiplied by 2. Ising model binary potentials 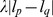 for 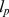 and 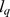 labels of pixels 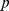 and 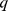 were assigned to neighboring pixels, using a 6-neighborhood in 3D. 20 random synapses in another training block were fully segmented and the 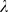 constant of the graph cut algorithm was selected by grid search, using the Jaccard index of the manually segmented synapses as a cost function. We used 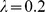 for our experiments. While high recall is more important for this step than high precision (we perform additional filtering later), the quality of the segmentation is important for the next step, which uses object-level features. In terms of recall, 16 test dataset synapses (7%) were missing in the set of the segmented synapse candidates. Graph cut segmentation of a 1.2 GByte block lasted 205 minutes.

Besides graph cut, we also experimented with hysteresis thresholding of the probability maps, using the detection threshold of 0.5 as the higher hysteresis threshold. While this segmentation approach is much faster, it often leads to under-segmentation and attaches long protrusions to the correctly detected objects, sometimes even connecting them to other nearby synapse candidates. Increasing the lower hysteresis threshold helps alleviate this problem, but leads to a drop in algorithm recall. In our best recall results with hysteresis thresholding 24 test dataset synapses were missing (10%). Unlike hysteresis thresholding, the graph cut algorithm has a “shrinking bias”, which makes it prefer shorter contours and avoid long protrusions. In case of synapse segmentation, this bias turns out to be beneficial.

For the final object classification step we selected two more training blocks from the data of [5], one with a fairly large extent in x and y (7478×7557×70 pixels), the other going through the whole volume in z (1024×1024×1233 pixels). Segmentation as described above was performed on the two blocks and the resulting synapse candidate objects were labeled as true or false. In total, 978 object-level labels were introduced with a similar number of positive and negative examples, avoiding objects which cross any of the boundary planes or image defects. These object labels and object-level features were used to train a Random Forest classifier [30]. Object feature computation and classification took 13 minutes on a 1.2 GByte block. Afterwards, the whole pipeline of pixel classification – segmentation – object classification was applied on the test dataset.

**Figure 1:**
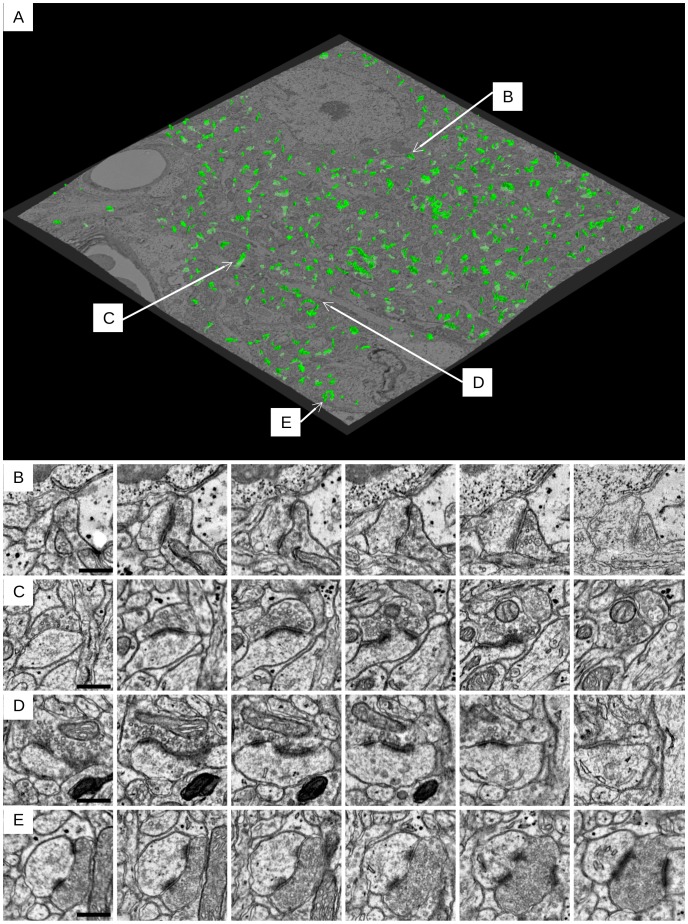
Synapses, detected in the test dataset. A: A 3D view of the synapses, detected in the test dataset. The detected synapses are shown in green. The central slice of the raw data is also shown for illustration (the shape distortion is caused by elastic stack registration). The data volume was downsampled by a factor of 10 in the x and y dimensions to show the full volume. B, C, D, E: More detailed synapse examples as an image series. Scale bars: 450 nm, every second slice is shown (distance between consecutive images is 90 nm).

Out of 238 synapses, the algorithm missed 28 (15 at the pixel classification stage and 13 at the object classification stage) synapses (11.7%) and additionally made 16 false positive detections (7%). A 3D view of the detected synapses is shown in Fig. 1. Increasing the object-level Random Forest threshold from 0.5 to 0.6 leads to higher precision: 20% false negatives, 2% false positives. Decreasing it to 0.4 leads to higher recall: 11% false negatives, 19% false positives. The errors of the algorithm were re-examined manually to ensure that these were not errors in the ground truth annotation. For reference, we also computed the error rate of the original human annotations, which were of different quality. The first annotator missed 9(4%) synapses and made 2 false positive detections, the second annotator missed 34(14%) synapses and made 4 false positive detections, the third annotator missed 29(12%) synapses and made 31 false positive detections.

## Discussion

Our results demonstrate that fully automated synapse detection is possible not only for isotropic FIB/SEM data, but also for anisotropic ssTEM data, and that a full pre-segmentation of the image stack into cells is not required for synapse detection. In fact, synapse detection can serve as pre-processing for cell segmentation and allow the segmentation effort to concentrate on those neuronal touches that actually form synaptic connections. Sparse tracing or skeletonization can be used in this case. A pre-detection of synapses would also allow to select specific neural processes for further analysis based on the properties of their synapses.

The proposed algorithm needs 6 hours to fully process a 1024×1024×1233 tissue block. Pixel classification is parallelized in ilastik, while the rest of the pipeline runs on a single core. The limited spatial extent and sparsity of synapses make synapse detection a very suitable task for blockwise processing. Adding a halo of 500 nm to each block would provide enough context information for all steps of the pipeline. The blocks can then be processed in parallel.

### Error Analysis

**Figure 2:**
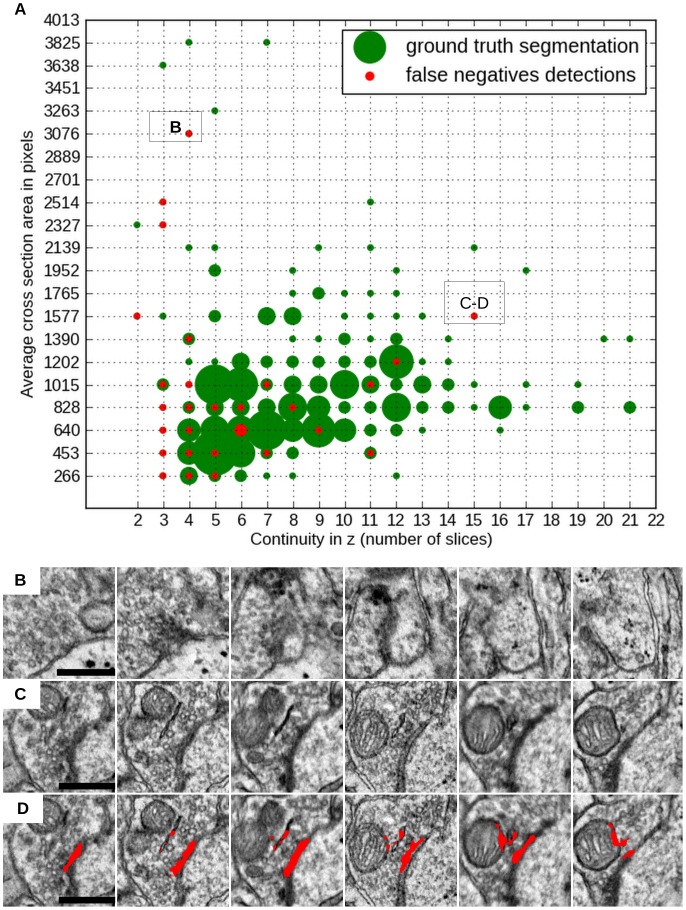
False negative errors. A - distribution of false negative errors as a function of the synapse average cross section area and continuity in z. For each bin of the 2D histogram its count is proportional to the radius of the displayed circle. Cross section area was measured in pixels and averaged across 5 central slices. B, C - serial sections of false negative detections. D – erroneous algorithm segmentation. All scale bars – 450 nm.

Detailed analysis of misclassified synapse candidates can highlight the shortcomings of the presented algorithm and show promising directions of future work. However, as for any classification algorithm that works in multidimensional feature space, errors of the Random Forest classifier are not always fully interpretable. In order to analyze the relation of synapse properties to the algorithm performance, we expanded our ground truth dataset from detections to approximate segmentations. We found that main factors, contributing to false negative detections, are small size in xy cross-section and lack of continuity along z (i.e. the synapse is only visible over a few z slices). The second factor affects not only physically small synapses, but also synapses oriented at a low angle to the cutting plane. Fig. 2(A) illustrates “detectability” of synapses, depending on their average area in xy (essentially, the size of the PSD in xy cross-section) and the number of tissue slices where they can be observed (z range). Two most outlying false negative detections are shown in more detail in Fig. 2(B) and Fig. 2(C–D). The large synapse in Fig. 2(C) is missed because of a segmentation error (segmentation is shown in red in Fig. 2(D)): it was erroneously connected to the nearby mitochondrion and smooth endoplasmic reticulum. The resulting object was then discarded by the object classifier. The synapse in Fig. 2(B), on the other hand, was missed already at the pixel classification stage. This example shows, that larger synapses can still be missed if they are only clearly visible in very few slices. In total, out of 28 synapses missed by the algorithm, 16 were also missed by at least one human annotator.

**Figure 3:**
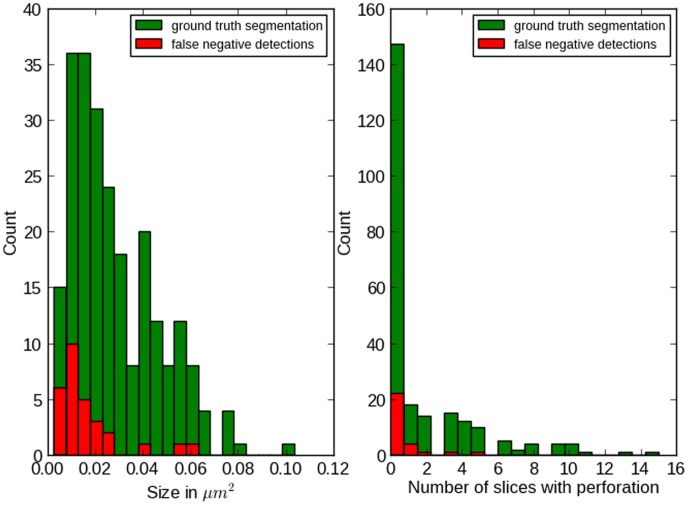
False negative errors against synapse size and perforation. Left: distribution of false negative errors as a function of synapse size (see text for details on size estimation). Right: distribution of false negative detections as a function of the number of slices, where the synapse is perforated.

Fig. 3 shows the distribution of false negative detections against two other factors: synapse size in 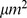 (left) and the size of the synapse perforation (right). The size in 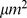 was computed by first finding the medial axis(“skeleton”) of the segmentation in each slice (using the scikit-image toolkit) and then summing up the lengths of the skeletons, multiplied by the slice thickness. No special treatment was applied to synapses parallel to the slicing plane (14 in this dataset), so their size is underestimated. The histogram in Fig. 3(left) again shows how difficult small synapses are: the second and third bin contain the same number of ground truth synapses, but the ones in the third bin are two times more “detectable”.

A very different factor is explored in Fig. 3(right). Here we counted for each synapse the number of slices in which it appears as two independent connected components. Apparently, perforated synapses are easier to find than non-perforated ones. This can be partially explained by their larger size, but in general it seems that synapses, perforated in two or more slices, can be detected quite reliably.

To summarize, the recall of automated synapse detection will most probably improve with the decrease in slice thickness. Increasing lateral resolution should also affect the performance positively. Tradeoff between resolution and imaging speed is an important decision in all connectomics experiments. We demonstrate, that for data with fairly average lateral and axial resolution (Anderson et al. in [6], [32] imaged the RC1 part of the retinal connectome at 2×2 nm lateral resolution, Briggman et al. in [4] imaged the retina by SBEM with 23 nm slices), the majority of the chemical synapses can be located automatically with a reasonably low false positive rate.

**Figure 4:**
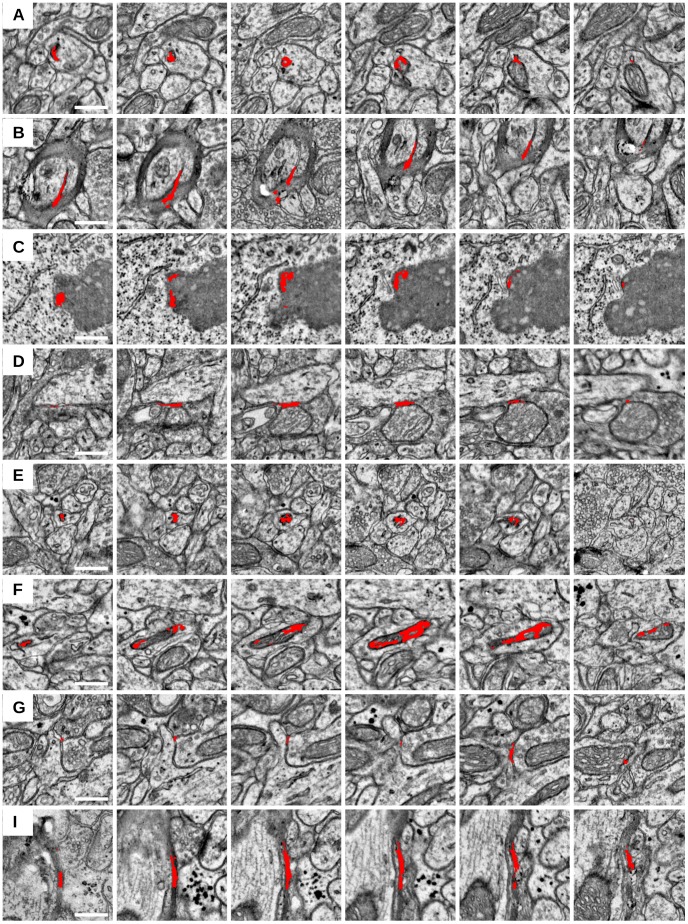
Examples of false positive detections. All scale bars – 450 nm, every second slice is shown (distance between consecutive images is 90 nm).

As for the false positive detections, the main sources of such errors are mitochondria, axon myelin sheaths and sites of membrane apposition without synaptic contact. The 16 false positive detections of the algorithm can be classified as follows: (i) apposition between the membrane of a mitochondrion and the membrane of the axonal bouton containing the mitochondrion (2 instances); (ii) myelin sheath or boundary between internal mesaxon of myelinating oligodendrocyte and the axon itself (3 instances); (iii) lysosome or degenerating mitochondrion (2 instances); (iv) outer membrane of a mitochondrion (2 instances); (v) spine apparatus (2 instances); (vi) mitochondrion and associated smooth endoplasmic reticulum (1 instance); (vii) border of a lipofuscin granule within a cell body (1 instance); (viii) apposition between small axon and dendrite, containing smooth endoplasmic reticulum oblique to the section plane (1 instance); (ix) segmentation error (1 instance); (x) membrane of axonal bouton apposed to dendritic spine neck and a second dendrite’s membrane, with bleed-through to the outer membrane of a mitochondrion contained within the second dendrite (1 instance). Some examples, along with the algorithm segmentations, are shown in Fig. 4. It is difficult to estimate how often a certain image structure could be mistaken for a synapse without first detecting all such structures in the test block. Instead, we roughly evaluated how many false positives from some of the classes above were misclassified as synapses by the first, high-recall, step of the algorithm and compared it to the final results, produced by the object classification stage. Outer membranes of mitochondria, sites of apposition of mitochondria and cell membrane and mitochondria with smooth endoplasmic reticulum were misclassified as synapses 160 times. Only five such cases could not be filtered out by object classification (examples in Fig. 4(A, D, F)). Membrane apposition sites without synaptic contact were misclassified as synapses 74 times at the first classification stage, only two remained after object classification (example in Fig. 4(G)). Spine apparatus was erroneously found 13 times by the first classifier, two cases were not corrected by the second stage (example in Fig. 4(E)). Nine false positives were first produced on the myelin sheaths or between myelin and mitochondria, 3 remained after object classification (examples in Fig. 4(B, I)).

### Outlook

While pixel classification can now achieve fairly high recall, it still misses some synapses. Perhaps, if such synapses were specifically sought and labeled, these results could be improved. For the object classification stage, some of the false positive detections would be removed if a reliable method existed for the segmentation of myelinated membranes. This is an interesting research problem in itself and we are currently working on it.

Since object classification is based on summary statistics of the segmented objects’ pixels, improvement of the segmentation accuracy would also lead to an improvement in detection. However, one has to keep in mind that more advanced segmentation methods usually involve higher-order potentials or more sophisticated pixel features and may not scale to Terabyte datasets. Since accurate segmentation of synapses is also very important for follow-up biological analysis, we plan to focus on this issue in the future, along with detection and counting of vesicles.

Image defects will probably become one of the major hurdles for the application of the presented algorithm to a large experimental ssTEM dataset. Even small image defects can hinder the classifier performance, as they unpredictably alter the grayscale distributions of the objects and their neighborhoods. Missing or damaged slices, as well as slices with rips and tears, have to be detected and somehow accommodated by the algorithm. Our test dataset includes 8 synapses located close to small image defects (but not overlapping with them). All 8 were detected correctly. Moreover, 8 image defects were misclassified as synapses by the first classification stage, all of them were correctly filtered out by object classification. However, additional preprocessing, including defect detection and inpainting, still has to be developed before processing the full dataset of [5].

We expect the proposed tool to be useful for neuroscience studies which involve analysis of synapse distribution and properties, as well as for the reconstruction of neural circuits, whether manual, automated or semi-automated. While the algorithm accuracy is not perfect, we believe it can serve as a pre-processing step to save the human expert the effort of ad hoc chemical synapse detection. It can also limit neural tracing to connections with interesting morphological properties (which have to be determined by human inspection of algorithm results).

## Materials and Methods

### Data Acquisition and Ground Truth Annotation

The test dataset of 7197×7351×64 pixels was cut out of the data, publicly provided by Bock et al [5] through the Open Connectome project (www.openconnecto.me). The test dataset was centered at approximately the following point in the hosted 10 TB dataset: (z = 507, x = 43247, y = 77492, zoom-level = 3!). It was re-registered with Elastic Align and Montage plugin for Fiji. The parameter settings for the alignment were as follows: layer scale = 0.07505; search radius = 133 px; block radius = 666 px; resolution = 2016; minimal PMCC r = 0.1; maximal curvature ratio = 10; maximal second best r/best r = 1; use local smoothness filter = true; approximate local transform = Affine; local region sigma = 1332 px; maximal local displacement (absolute) = 33 px; maximal local displacement (relative) = 3.00; test maximally = 8 layers; maximal iterations = 1000; maximal plateau width = 200; stiffness = 0.1; maximal stretch = 2000 px.

Three independent human annotators (trained by an expert neuroscientist, but themselves inexpert) then marked all synapses in the rectangle [[1000, 1120, 30], [6000, 6120, 36]], using the FiJi TrakEM2 plugin [33]. The three annotation sets were compared automatically and an expert neuroscientist resolved all cases where the three annotators did not agree. The resulting test set contained 238 synapses.

### Automated Detection Algorithm

**Figure 5:**
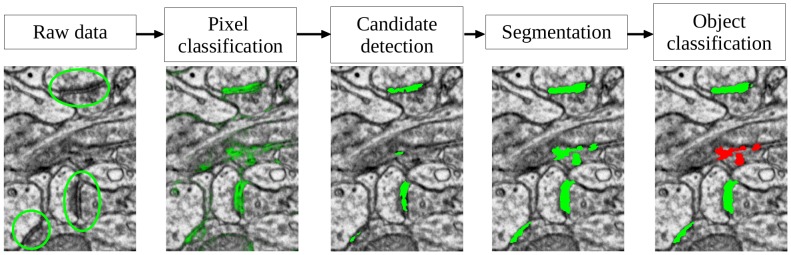
The proposed synapse detection pipeline. Left to right: raw data with 3 synapses, shown in green circles; probability map of the synapse class; detection results; graph cut segmentation results; object classification results, with positively classified objects shown in green and the negatively classified object in red.

Our goal in designing the algorithm has been to provide a performant synapse detector with the potential to scale to very large datasets at a low human interaction cost. The algorithm is based on two-stage learning, where pixel classification and graph cut segmentation are used for high recall synapse candidate detection and the second learning step is used to filter the candidates based on their object-level features in order to improve the precision. Fig. 5 illustrates the described pipeline. In terms of user annotation time, the main burden lies in the annotation for object classification, while the pixel classification, like in [24], operates on a very sparse set of labels. Object labeling is much faster and less strenuous than pixel labeling, as most potential synapses are highlighted and adding a label only requires one click per object.

#### Pixel classification

**Figure 6:**
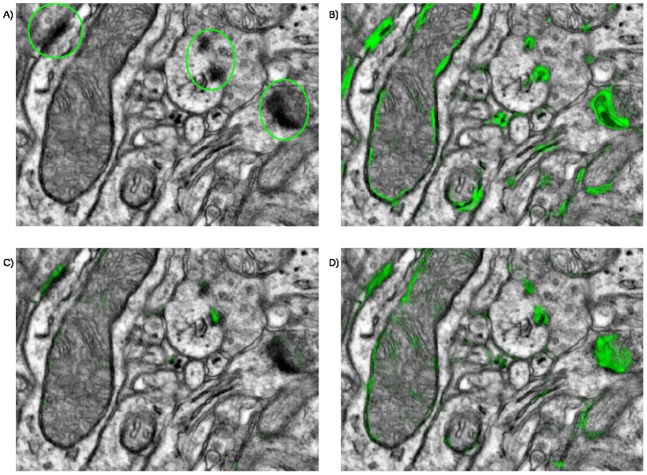
Benefits of 3D processing with upsampling. A: raw data, with three synapses in green circles. B: probability map of the synapse class after classification with 2D features – low precision of the prediction. C: same for classification with 3D features without upsampling – low recall. D: same for classification with 3D features and upsampling by 2 along the z axis. The remaining false positives are filtered out by the object classification step.

Most algorithms proposed for automated ssTEM data analysis perform pixel classification in 2D and then link the predictions along the z-dimension. We, on the contrary, propose to perform the classification directly in 3D, thus taking more axial context into account. In our experiments this clearly improves the precision of the algorithm without much loss in recall (Fig. 6(B) compared to Fig. 6(D)). The remaining false positives are filtered out by the object classification procedure described in the next paragraphs. Additionally, we interpolate the images by a factor of two along the z-dimension to partially make up for the anisotropic resolution of the data. This improves the performance of nonlinear image filters [34] and increases the recall of the algorithm (Fig. 6(C) compared to Fig. 6(D)). We also experimented with anisotropic image features with a smaller filter extent in z and with higher interpolation factors, but these did not improve the results significantly.

Features were selected according to their Gini importance as measured by the Random Forest classifier. We used the ilastik learning and segmentation toolkit [35] for interactive pixel classification and offline prediction on the test dataset. Internally, ilastik uses the C++ vigra image processing library for classification and image feature computation. While some of the feature scales are different, the pixel classification procedure is very similar to the one described in [24]. The full list of features can be found in Table 1.

**Table 1:** Pixel features.

Feature	Sigmas	# of channels
Intensity of the Gaussian-smoothed image	0.7, 1.0, 1.6, 3.5, 5.0, 10.0	1
Eigenvalues of the Hessian matrix	1.6, 3.5, 5.0, 10.0	3
Laplacian of Gaussian	3.5, 5.0, 10.0	1
Difference of Gaussians	5.0, 10.0	1
Eigenvalues of the structure tensor	5.0	3
Total number of features		26

Features, used for pixel classification. All features were computed in 3D. The “Sigmas” column lists the sigmas of the Gaussians, used for smoothing the data (same sigma was used for all 3 dimensions). For the eigenvalues of the structure tensor, the second scale parameter was set to sigma/2, for the difference of Gaussians the second Gaussian sigma was set to 0.66*sigma.

### Detection and segmentation

To detect synapse candidates in pixel probability maps produced in the previous step, we threshold the synapse probability map at 0.5, run connected components analysis and discard very small (less than 100 pixels in our test dataset) and very large (more than 1000000 pixels) connected components. For the remaining components we extract the bounding boxes and enlarge them in such a way that the enlarged bounding box can be assumed to contain the full extent of the detected synapse (500 nm according to [36]). In each enlarged box a synapse candidate is then segmented using the standard graph cut algorithm [31]. Following [31], define a segmentation 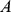, which assigns labels 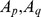 to pixels 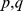. The segmentation problem is then formulated as the minimization of the following energy function: 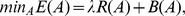 where 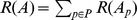 is the so-called regional term and 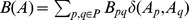 is the boundary term. 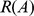 expresses the conformity of the pixels to their assignment. We use the synapse probability of the pixels, multiplied by 2. The boundary term expresses the continuity of objects and favors assigning the same label to neighboring pixels. 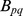 function assigns a higher cost to inconsistent labeling of similar neighboring pixels and relies on some measure of pixel similarity, such as local intensity gradient. We use the standard 3D 6-neighborhood to define neighboring pixels and a constant 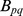 term, which does not depend on the underlying pixel values like the boundary term in Conditional Random Field models. The reason for this choice of 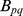 is that a true synapse object contains two strong edges, which form a synaptic cleft, so the algorithm should not additionally encourage object boundaries to coincide with strong edges in the underlying image. The region term is also negatively influenced by these strong edges and can not counterbalance the contribution of the boundary term. Our choice for performing the segmentation in a loop over the potential objects rather than globally is motivated by the reasons of scalability. Graph cut can be computed in low-order polynomial time. A prior detection would make the segmentation procedure scale linearly with the number of detected objects, which are fairly sparse, and not polynomially with the number of pixels in the 3D volume. Besides, segmentations of individual synapse objects are in principle independent of each other. The segmented objects are taken as synapse candidates and additionally filtered in the next classification step.

#### Synapse candidate classification

Two types of object features were considered for classification: features of the pixels of the object itself and features of the object neighborhood, excluding the object. The neighborhoods of the objects were computed using anisotropic 3D distance transform and included points within 30 pixels in x and y and 1 pixel in z from the object. The feature accumulator framework of the vigra image processing library was used to compute the features efficiently. Given a labeled connected components volume, the framework allows to compute all pixel statistics for all connected components in one run, using as many sweeps over the data as the selected highest-order statistic requries. The features can be computed directly on the pixel grayscale values, on the object coordinates (bounding boxes, etc) and on the projections on the objects’ principal components. Blockwise computation with subsequent merging is available for most features as well. The histograms of local binary patterns [30] were computed using the scikit-image library. The full list of features and their parameters can be found in Table 2. As before, predictions were performed using the vigra implementation of the Random Forest classifier with 100 trees grown to purity.

**Table 2:** Object features.

Feature	Parameters	# of channels
Mean		1
Mean, object neighborhood	Neighborhood size: (30, 30, 1)	1
Variance		1
Variance, object neighborhood	Neighborhood size: (30, 30, 1)	1
Skewness		1
Skewness, object neighborhood	Neighborhood size: (30, 30, 1)	1
Kurtosis		1
Kurtosis, object neighborhood	Neighborhood size: (30, 30, 1)	1
Local binary patterns’ histogram	P = 8, R = 1, method = ‘uniform’	10
Local binary patterns’ histogram, object neighborhood	Neighborhood size: (30, 30, 1)	10
The ratio of the largest principal component to the smallest		1
Total number of features		29

Features, used for object classification. The size of the neighborhood is specified in pixels for x, y and z dimensions. The neighborhood was computed using anisotropic distance transform in 3D.
